# Gene therapy advances using canine and feline animal models of inherited retinal degeneration

**DOI:** 10.1038/s41433-025-03825-y

**Published:** 2025-06-03

**Authors:** Simon M. Petersen-Jones, Billie Beckwith-Cohen

**Affiliations:** Department of Small Animal Clinical Sciences, College of Veterinary Medicine, Michigan State University, East Lansing, MI 48824 USA

**Keywords:** Outcomes research, Eye diseases

## Abstract

Inherited retinal degenerations (IRDs) are a genetically heterogenous group of visually impairing conditions that affect many people worldwide. They are caused by mutations in a variety of genes with a range of vision loss onset from childhood to middle-age. Many IRDs are inherited in an autosomal recessive fashion and are due to loss of function of the gene product, allowing for a standard gene augmentation approach in which a normal copy of the mutated gene is introduced. Retinal gene delivery using adeno-associated viral (AAV) vectors has proven to be the safest and most effective approach and has been used in many clinical trials. Introducing a normal copy of the mutated gene is applicable when used prior to advanced photoreceptor degeneration while there are still sufficient “rescuable” photoreceptors. Several naturally occurring IRDs which are homologous to human IRDs have been identified in the dog and cat. A subset of these has been successfully used for preclinical trials that have contributed to regulatory approval for subsequent human clinical trials. While most have been for recessive conditions due to loss of gene function, examples of dominant disease requiring a knockdown of the mutant transcript exist and have been used. IRDs bear a considerable economic and societal impact. Identification and familiarity with appropriate models that can lead to successful therapeutic approaches are of great significance. This narrative review aims to summarize advances in gene therapy using canine and feline models for human IRDs and discuss their advantages and disadvantages as well as future perspectives using them.

## Introduction

Inherited Retinal Degenerations (IRDs) are a heterogenous group of visually impairing conditions. Thus far, mutations in over 300 genes have been associated with IRDs (RetNet, https://RetNet.org/). Gene augmentation therapy, introduces a functional gene copy to nondividing retinal cells, offering a potential cure for autosomal recessive IRDs. The long-term episomal persistence of the transgene offers sustained rescue [1] meaning that a single treatment should be sufficient. However, as the treatment is invasive and permanent, any intervention needs to be preceded by preclinical and clinical trials assuring that no permanent harm will be done, and that the treatment is likely to succeed. The eye and retina within it are easily accessible and therefore practical targets for gene augmentation therapy. Challenges associated with development of novel gene therapies for IRDs are associated with availability of suitable models that recapitulate the human disease, the mode of inheritance and mode of vector delivery. Small animal models such as rodents and zebrafish, and cell-based model systems have contributed greatly to the advancement of the field. These model systems are cost effective and can be easily manipulated. Nonetheless, the complexity of the human visual system often necessitates similarly complex model systems for dependable conclusions, prior to introducing gene therapy.

Initial proof-of-concept studies in dogs with a mutation in the *RPE65* gene, a model for Leber Congenital Amaurosis type 2 (LCA2), were essential in developing the first FDA-approved gene augmentation product (voretigene neparvovec-rzyl; Luxturna®) and the only gene therapy product approved for an IRD [2]. The rapid advancement of gene therapy from a dog model to human clinical trials was made possible due to the availability of a spontaneous canine model and anatomical features shared by these species. Dogs have large eyes with similar optical and neural structures to humans, including regions of high cone density, and binocular vision. The success of Luxturna in offering vision to previously blind children led to further experiments with a similar goal: replacing defective genes and restoring vision. Dog models have since been used for development of several gene therapy products currently undergoing clinical trials. This review will cover the use of spontaneously occurring IRDs in dogs and cats to develop and test gene therapy products. Table 1 summarized these models, along with the stage of preclinical and clinical investigation. Advantages and disadvantages of these models and their alternatives over other large and small animal model systems will be discussed.

**Table 1:** Dog and cat IRDs - gene therapy in human clinical trials.

Gene	Function	Species	Inheritance	Stage of development of human therapy and clinical trial number
**Leber congenital amaurosis**				
*RPE65*	Visual cycle	Dog	AR	FDA approved product - Luxturna
*NPHP5*	Photoreceptor cilia formation and trafficking	Dog	AR	Clinical trial planned
**Retinitis Pigmentosa**				
*PDE6A*	Rod phototransduction	Dog	AR	Clinical trial- NCT04611503
*PDE6B*	Rod phototransduction	Dog	AR	Clinical trial- NCT03328130
*CNGB1*	Mediates rod Phototransduction	Dog	AR	Clinical trial planned
*RHO*	Photopigment	Dog	AD	Preclinical
*RPGR*	Exact function unknown	Dog	XL	Clinical trials- NCT03116113; NCT03252847; NCT03316560; NCT03584165; NCT04312672; NCT04517149; NCT04671433; NCT04794101; NCT04850118; NCT05874310; NCT05926583; NCT06275620; NCT06333249; NCT06492850; NCT06646289
**Other IRDs**				
Achromatopsia -*CNGB3*	Mediates cone photoresponses at bright light	Dog	AR	Clinical trials-NCT02599922 NCT03001310, NCT03278873
Best disease – *BEST1*	Anion transport and intracellular calcium regulation	Dog	AR	Preclinical
cCSNB - *LRIT3*	Synaptic formation and assembly in OPL	Dog	AR	Preclinical
Cone-rod synaptic disorder – *CaBP4*	Modulate synaptic Ca in OPL	Dog	AR	Preclinical
Fundus albipunctatus – *RDH5*	Visual cycle (biosynthesis of 11-*cis* retinaldehyde)	Cat	AR	Preclinical

## Methods

**Fig. 1:**
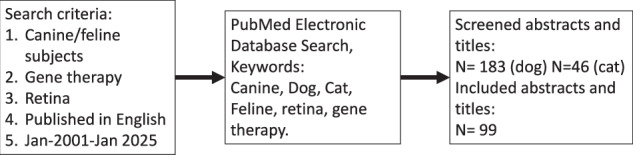
Literature search strategy.

A narrative review of the literature was conducted. The SANRA, Scale for the Assessment of Narrative Review Articles, was used as a guide in writing this manuscript [3]. An extensive PubMed database search was performed to identify English-language published articles that reported on dogs and retinal gene therapy. The search was inclusive of publications between January 2001 and January 2025. The year 2001 was chosen as it was when the first dog, Lancelot, was treated with gene therapy for a mutation in *RPE65* [4]. Figure 1 presents a flow chart summarizing the search strategy. Eligible studies were published in the aforementioned timeframe in English. They were peer reviewed clinical, observational, interventional or review studies. Review studies were primarily used to ensure that no pertinent study was overlooked. Peer reviewed conference proceedings were considered if published within the last 5 years. Publications were excluded if they studied methods for gene therapy using experimental models in wildtype animals or in non-spontaneous or induced retinal disease. Studies addressing retinal manifestations of systemic disease such as lysosomal storage diseases were also excluded [5, 6]. While all included studies were reviewed, due to redundancy, citations were only made when needed. One manuscript was available both as preprint, and in final form and the preprint was not included. The search query used was: “((dog OR canine) AND (retina*)) AND ((gene therapy) OR (gene augmentation therapy)) AND ((english[Filter]) AND (2001:2025[pdat]))”.

The search was repeated replacing the term “dog” for “cat” and “canine” for “feline”.

## Mammalian IRD models

Over 90% of the mouse and human genomes can be partitioned into regions of conserved synteny [7]. Mice are excellent models for initial studies to characterize and develop therapeutics for IRDs because of the ease of genetic manipulation, relative speed of producing affected animals and lower associated costs. For these reasons mice, and to a lesser extent rats, have served as preferred model organisms to study human disease [8]. Nonetheless, some important phenotypic and genotypic differences exist making mouse models suitable to study some, though not all human diseases [8]. While all mammalian species share the same basic retinal features, rodents lack a retinal region of high photoreceptor density, particularly cone density. The human macula is cone-dense and is used for high-acuity colour vision. Several IRDs preferentially affect the macula and therefore cannot be fully modelled by rodents. Additionally, humans and dogs have structures called calyceal processes at their photoreceptor inner/outer segment junction [9], a site of expression of certain IRD genes. As rodents do not have a calyceal processes as part of their photoreceptors they may not be good models for IRDs affecting this structure [10]. Further, despite a similarly between mammalian genomes, certain IRD genes such as *EYS*, are not present in the rodent genome. In other cases, despite genotypic homology, mutations in mouse (or rat) models either do not result in a phenotype or the phenotype does not closely resemble the human condition. These disparities and difficulty in identifying orthologous phenotypes can be associated with variations in gene expression, epigenetic variations, and dissimilarities between human and murine immune systems [8]. Lastly, while retinal similarities are extensive, the development of the visual pathways and cortex are considerably different between mice and large animal model systems. The human and mouse visual systems share a general anatomic resemblance but vary greatly in complexity. Mice have a minimal region of binocular vision, and therefore lack ocular dominance columns and complex binocular representation as seen in humans. A study in mice showed that effects from the AAV vector used was observable in the contralateral eye [11]. Though clinically beneficial, this is unlikely to be the case in large mammals such as dogs and people, where untreated eyes are not reported to show improvement. The binocular benefit complicates the use of mice in studies that require a within animal control. While functional *electrophysiologic* recovery in the level of the retina can be readily studied in mice, the implications for functional *vision* recovery cannot be readily translated to people.

The main additional large animal models used for IRD studies include the pig, sheep and non-human primate (NHP). Pigs are suitable because of a similar eye size to humans and the cone-rich visual streak [12]. Their genome has been modified to create new models with human relevant mutations. Several models have been generated including ones with a knockin of the common human rhodopsin mutation Pro23His [13], and knockouts of *ABCA4* to model Stargardt disease and of different genes linked to Usher Syndrome [12, 14, 15]. Disadvantages include behavioural and logistical challenges in animal handling for procedures such as routine eye examinations. In particular, adult models using a full-size pig background that may weigh 100 kg or more presents challenges in handling the animals. Sheep with spontaneous mutations in *CNGA3* have been identified. Successful gene augmentation in these sheep has shown long-term maintenance of the rescue has contributed to the establishment of human clinical trials [16–18].

NHPs are the closest available model to study human IRDs since they have a macula and fovea. There are a limited number of spontaneous IRDs in NHPs and while it is now possible to produce models by genome editing [19, 20] the process is expensive. While the advantages of NHP models are obvious, the slow reproductive rate and long expected life span can limit their utility for studying therapies for IRDs. Further challenges are associated with high costs, limited facilities approved for handling NHPs, zoonotic potential and ongoing resistance stemming from ethical concerns.

For these reasons there has been an increased need for suitable animal models to study and develop treatments for IRDs, and this need is well met by using dogs and cats with spontaneous IRDs.

### Important features of the dog and cat models

**Fig. 2:**
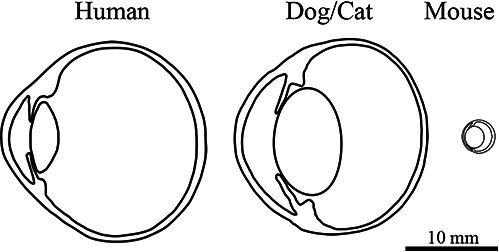
Montage comparing globe dimensions between human dog/cat and mouse (adapted from Petersen-Jones [102]).

**Fig. 3:**
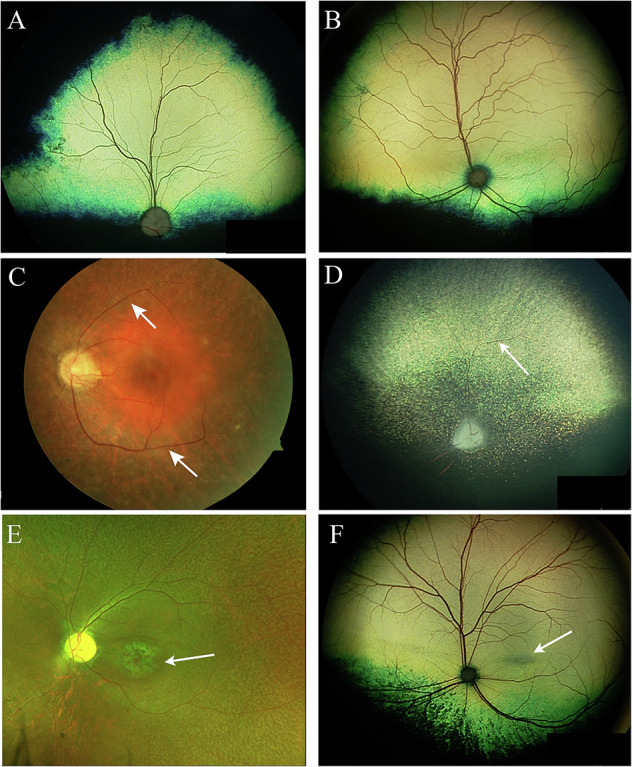
Comparative colour fundus images from dogs, cats and people. Colour fundus images of normal dog (**A**) and cat (**B**). Note that both species have a tapetum. This structure is positioned in the inner choroid. **C**, **D** Show a human and a dog respectively with *CNGB1* mutations. The arrows indicating attenuated blood vessels in both human and dog. **E**, **F** Are human and cat respectively with mutations in *RDH5*. The arrows point to degenerate macula in the human patient and degenerate area centralis in the mutant feline. Image **C** is modified from Petersen-Jones et al. [61]. Image **E** is modified from Occelli et al. [90].

Dogs and cats share both anatomic and physiologic resemblance to the human eye and visual system. The dimensions of the globe and its intraocular compartments are similar to that of humans (Fig. 2). This resemblance in globe size allows for similar injection, implant and surgical procedures to be used in these species comparable to the interventions that will be considered in humans. Figure 3A, B shows normal wide angle colour fundus images of dog and cat respectively. Note that unlike humans both species have a tapetum which is a coloured reflective structure within the inner choroid.

Dogs and cats have a cone rich region called the visual streak which serves for higher-acuity vision [21, 22]. Temporally within the visual streak lies the area centralis, the region that has the highest density of cones. Studies in the dog showed that peak cone density in the centre of the area centralis is comparable to the peak cone density of macaque and human retinas, making it analogous to the human macula [23]. While neither dogs or cats have a fovea, dogs have been described to have a unique focus of high cone density within their area centralis, which was termed the foveal bouquet [23]. While dogs and cats do not have the same visual acuity that people do, dogs in particular are easily trained to test visual acuity using behavioural tasks [24–26]. Their Snellen visual acuity varies by test and study, but is up to 20/60 [26], which is far superior to the mouse that has a visual acuity of 20/1648 [27]; a value which would be considered legal blindness in people. Dogs and cats also rely on a large binocular region compared to mice. While binocular overlap is inferior in comparison to humans, both species have binocular competition, and have been used to study amblyopia and development of the visual system [28].

Discovery of pathological ocular (and other) phenotypes in dogs has been largely aided by routine screening of purebred dogs, and practices of pedigree breeding which have led to an increase in the prevalence of naturally occurring, non-lethal recessive mutations. Such mutations are further propagated by practices of consanguineous breeding, which are particularly common in rare breed populations. This has also occurred in cats, but to a lesser extent. While some IRDs have been sporadic and rare, others may occur commonly within specific breed populations. IRDs in dogs and cats often mimic IRDs caused by mutations in the analogous genes in humans. Disadvantages of dog and cat models is that those available are only spontaneously occurring mutations. There are no “engineered” models meaning that a model with a specific mutation or with a mutation within a specific gene might not have been identified in screening the pet population. Establishment and maintenance of a colony of mutant dogs and cats is expensive, and breeding takes time. There are limited centres that are set up to maintain such colonies. Lastly there are strong ethical considerations when using companion animal species for experimental studies and in some countries it is difficult, if not impossible, to carry out such studies even when the dog or cat model maybe the only model that faithfully recapitulates the human condition.

### Examples of specific IRDs in dog and cat models used for therapy development

Table 1 lists the cat and dog models for which preclinical gene therapy has been reported and lists the human clinical trials underway for each gene.

#### Leber congenital amaurosis models

##### RPE65

Mutations in *RPE65* (retinoid isomerohydrolase RPE65) are a cause of severe childhood-onset visual impairment known as Leber congenital amaurosis type 2 (LCA2). A mutation in *RPE65* was identified in the Briard breed resulting in a phenotype similar to LCA. The *RPE65* mutant dog was important in providing the first proof-of-concept gene augmentation therapy for LCA2 [4]. The FDA approved product Luxturna® (approved in 2017 by FDA and in 2018 by EMA) was the first gene augmentation product to be approved and remains the only gene therapy product approved for an IRD [2]. The dog model was used by several groups to answer questions pertaining to therapy such as whether the second eye could be successfully treated without interference of immune responses to the vector/transgene [29], and whether the therapy completely halted retinal degeneration [30]. Patients treated with Luxturna® reported remarkable improvement in quality of life, as well as improved visual function outcomes [31]. Nonetheless, it is important to recognize that the dramatic improvement in retinal function in the canine model as shown by electroretinography [32] has not been matched in human subjects treated with Luxturna®, perhaps indicating species differences. Some studies reported continued degeneration and photoreceptor loss despite treatment. This was noted both in dogs treated later stages of the diseases, and in human RPE65-LCA, indicating that in this disease treatment alone might not prevent photoreceptor degeneration in the long-term [30]. Also, once in the clinic some adverse effects were noted due to gene therapy associated uveitis and perifoveal chorioretinal atrophy [33–35]. The dog model can show similar gene therapy associated retinal changes and continues to offer an opportunity for further study.

##### NPHP5

*NPHP5* (nephrocystin 5) mutations cause recessive LCA in people. This can be syndromic, with accompanying cystic kidney disease (Senior-Løken Syndrome) [36]. A dog model with a frame-shift mutation in *NPHP5* [37] was identified and shown to have a phenotype only involving the retina and characterized by early-onset photoreceptor degeneration but with area centralis preservation of non-functional cones [38]. Proof-of-concept gene augmentation therapy studies were performed using this model and showed that early intervention could halt rod degeneration and allow for cones to develop functional outer segments [39]. The rescue was maintained long-term [40]. The success of gene augmentation therapy in the dog model led to *NPHP5*-LCA to be one of 8 gene therapy projects to be taken through to a clinical trial under the Bespoke Gene Therapy Consortium, a private/public consortium headed by the Foundation of the NIH [41].

##### RPGRIP1

*RPGRIP1* (RPGR Interacting Protein 1) mutations in people most commonly result in LCA type 6, although a cone-rod dystrophy phenotype is also reported. A colony of Miniature Longhaired Dachshunds with a relatively severe cone-rod dystrophy was established. Genetic studies showed affected dogs were homozygous for an insertion in *RPGRIP1* [42]. Gene augmentation to introduce a normal copy of *RPGRIP1* was subsequently performed on dogs from the closed colony with positive results [43]. Subsequently animals outside of the colony with the same *RPGRIP1* insertion that failed to develop photoreceptor degeneration were identified. Studies of these dogs showed there was second locus contributing the photoreceptor degeneration and this was shown to be a deletion involving *MAP9* [44]. This variant appears to be acting as a modifier, and is necessary for the *RPGRIP1* mutant dogs to develop the cone-rod dystrophy phenotype. A potential third interacting locus in the dog further complicates the situation [45].

#### Retinitis pigmentosa models

Mutations in several genes have been identified in pet dogs with the canine equivalent of RP, in veterinary medicine the term progressive retinal atrophy is used to describe these conditions [46]. Some of these have been established as colonies to allow more detailed study and development of gene therapy. Most mutations cause recessive disease, however an opsin mutation has been identified causing dominant disease and different *RPGR* mutations causing X-linked disease.

##### PDE6A

*PDE6A* (Phosphodiesterase 6A) mutations in humans result in a recessive RP [47, 48]. A frame-shift mutation in *PDE6A* was identified in the Cardigan Welsh Corgi breed of dog [49]. Characterization, after establishing a colony, showed that there was a failure in maturation of rod outer segments followed by a rapid loss of rods associated with accumulation of high levels of cGMP which occurs due to the complete failure of rod phototransduction [50]. The cone outer segments are also stunted and the cones secondarily die as the supporting rods are lost. The lack of PDE6A results also in a lack of PDE6B in rods, as PDE6A may be required for the trafficking of PDE6B to the rod outer segment. Gene augmentation therapy rescues the phenotype when performed in young puppies treated between 1 and 3 months of age [51]. The treated dogs showed maintenance of rod photoreceptor rescue and resulting preservation of cones for up to 5 years post injection. The vector for these studies was an AAV8 capsid packaged with the human *PDE6A* cDNA under control of a human rhodopsin promoter. A similar construct was used in human clinical trials (NCT04611503). Initial reports were of reduced BCVA and in some patients foveal thinning [52].

##### PDE6B

*PDE6B* (Phosphodiesterase 6B) mutations result in a recessive RP [53]. A mutation in *PDE6B* was identified in an Irish Setter dog model of RP that had already been the subject of detailed characterization [54]. Gene therapy in the dog model has been reported to restore rod function and preserved function and structure in the treated region for up to 3.5 years [55, 56]. A phase I/II human clinical trial of gene augmentation therapy showed safety and evidence of improved rod function [57].

##### CNGB1

Mutations in *CNGB1* (Cyclic Nucleotide Gated Channel Subunit Beta 1) are an important cause of RP. Sequencing data showed *CNGB1* to be the sixth most prevalent RP gene [58]. A mutation in *CNGB1* was identified in the Papillon breed of dog [59]. Successful gene therapy and development of a translatable AAV vector for *CNGB1*-RP using the dog *CNGB1* model have been published. *CNGB1*-RP patients have a slower rod loss than seen with other forms of RP (e.g. *PDE6A*-RP) providing a greater window of opportunity for gene augmentation [60]. The *CNGB1* mutant dog model recapitulates the slower rate of photoreceptor degeneration [59]. Figure 3C, D show fundus images of human (3C) and dog (3D) with *CNGB1*-RP. The slower progression of rod loss coupled with the relative prevalence of *CNGB1*-RP makes it an attractive target for future clinical trials. Gene augmentation studies in both knockout mice and the spontaneous dog model restored rod function and slowed photoreceptor degeneration [61–63]. *CNGB1*-RP was one of the 8 gene therapies (including *NPHP5*- LCA as mentioned above) to be selected for a human clinical trial under the Bespoke Gene Therapy Consortium [41].

##### Rhodopsin

Rhodopsin mutations are an important cause of RP. Most commonly mutations result in dominant RP. The disease mechanisms of many of the described human mutations have been elucidated [64]. A mutation in rod opsin was identified in dogs and causes a dominant degeneration, similar to the majority of human opsin-RP forms [65]. The retina of the affected dog is very sensitive to light exposure [66]. Similar mutation classes in human rhodopsin-RP patients are hypothesized to have degeneration accelerated by light exposure [64]. The mutant rhodopsin product has an adverse effect on the retina (dominant negative effect). Therefore, a strategy to knock down the mutant allele and replace it with a hardened rod opsin cDNA resistant to the knock down is required [67].

The knockdown replace approach has not entered human clinical trials. However, other strategies such as antisense oligonucleotides (NCT04123626) and a gene editing approach (NCT05805007) have been assessed in clinical trials.

##### RPGR

*RPGR* (Retinitis Pigmentosa GTPase Regulator) plays an important role in the photoreceptor cilium. Mutations in RPGR cause X-linked RP which is a particularly severe form of RP affecting young men. Dogs with two different spontaneous RPGR mutations have been identified. A microdeletion is responsible for one of the canine forms and a missense mutation for the other [68]. The phenotype differs in rate and topographical differences in photoreceptor loss between the two forms [69].

Several studies with canine *RPGR* mutant models are reported using different constructs [69, 70]. Human clinical trials are underway by different groups of investigators (see Table 1 for the ClinicalTrials.gov identifiers (NCT numbers) for the trials) see McClements et al for a summary [71]. Some results have been published with one study showing visual field improvements in 6 of 18 treated patients [72] and another study reporting improvements in retinal sensitivity and functional vision [73].

#### Other retinopathies

##### Congenital Stationary Night Blindness

Congenital stationary night blindness usually results from mutations that involve genes important for photoreceptor to bipolar transmission or bipolar function. A spontaneously occurring mutation in *LRIT3* (Leucine Rich Repeat, Ig-Like and Transmembrane Domains 3) was identified in beagle dogs and serves as a model for the Schubert-Bornschein form of cCSNB [74, 75]. Affected dogs have an absence of rod b-wave with cones showing loss of ON bipolar cell (BC) response but a preservation of OFF-BC activity which allows them functional cone-mediated vision. The condition was stationary with no retinal degeneration. Gene augmentation therapy in the dog model was successful in restoring function (resulted in ERG b-wave being restored and rod-mediated vision [76, 77].

##### Best disease

Bestretinopathies or Best disease are a heterogenous group of ocular diseases caused by mutations in the *BEST1* gene. Most of the conditions in people show a dominant mode of inheritance with recessive forms being rare. In the dog, the forms identified have all shown recessive inheritance. The gene product is the bestrophin 1 protein and is located on the basolateral membrane of the RPE, and acts both as an anion channel, and a regulator of intracellular calcium [78]. Dogs have been reported to have three different mutations in *BEST1*, which have been associated with a specific clinical phenotype called Canine Multifocal Retinopathy (CMR) 1, 2 and 3 [79, 80]. The affected dogs have regions of retinal elevation and also microdetachments, thus mimicking the human disease. Preclinical gene augmentation therapy using constructs with either the dog or human *BEST1* cDNA under control of a *VMD2* promoter were tested on the dog models. Therapy with both vectors reversed the characteristic disease lesions and also the microdetachments [81]. Subsequently a translatable vector with the human *BEST1* cDNA has been tested in IND-enabling studies in the dog model showing efficacy (reduction in retinal detachments and an increase in ERG amplitudes) and a NOAE (no observed adverse effect) dose and may lead to future clinical trials [82].

##### Cone-rod synaptic disorder

A spontaneously occurring mutation in *CaBP4* (Calcium binding protein 4) was identified in the Whippet breed of dog and is a model for human *CaBP4* related retinopathies [83]. In humans with *CaBP4* mutations the phenotype has been variably classified as an incomplete CSNB, LCA and cone-rod synaptic disorder. CaBP4 associates with C-terminal domain of Ca(V)4.1 alpha 1 and shifts the activation of the channel to hyperpolarized voltages [84]. The affected dogs lack an ERG b-wave for both rods and cones [85]. In contrast to the apparent stationary nature of human cone-rod synaptic disorder the canine condition does lead to a progressive photoreceptor degeneration. There is an immaturity of photoreceptor ribbon formation and lack of expression of key synapse genes. Gene augmentation therapy prior to complete photoreceptor degeneration restores the rod and cone mediated ERG b-wave and allows for maturation of synaptic ribbons and normalizes levels of key synaptic proteins [83].

##### Achromatopsia

Mutations in several cone photoreceptor genes can result in achromatopsia, which is manifest as a loss of daytime vision. One such gene is *CNGB3* (Cyclic Nucleotide Gated Channel Subunit Beta 3). CNGB3 is one of the subunits that makes up the cone cyclic GMP gated channel which is essential for cone phototransduction. A mutation in *CNGB3* was identified in dogs [86]. Two forms were identified, one resulted from a genomic deletion involving *CNGB3* and was identified in the Alaskan Malamute breed of dog and the other was a missense mutation at a well-conserved residue and was identified in the German Shorthaired Pointer breed of dog. The phenotype of dogs with each mutation type was of a congenital loss of cone function and day blindness. Subsequently colonies of affected dogs with both mutations were used in gene therapy trials. When younger dogs were treated by subretinally delivered AAV vector cone function was restored [87], however in older dogs the therapy did not restore function. Interestingly treatment with ciliary neurotrophic factor (CNTF), which causes an initial loss of outer segments (described by the authors as photoreceptor deconstruction) followed by regrowth allowed the AAV gene augmentation therapy to restore a cone ERG and cone-mediated vision [88]. There are different clinical trials underway in patients with achromatopsia due to *CNGB3* mutations. Results from one of the human clinical trials is published showing a typical safety profile for subretinal delivered AAV therapy. While the study showed some indications of functional improvements it raised concerns that lack of cortical plasticity could be a limiting factor for visual recovery [89].

##### Fundus albipunctatus

Fundus albipunctatus is most commonly the result of mutations in *RDH5* (retinol dehydrogenase 5). Affected patients have a congenital night blindness, some degree of cone abnormality, which in some instance progresses to debilitating macular degeneration (Fig. 3E). Multiple white spots appear across the fundus giving the name Fundus Albipunctatus. RDH5 is the last enzyme in the classic visual cycle responsible for regeneration of 11-*cis*-retinal for transport to the photoreceptors for regeneration of the visual pigments. Other RDHs play a role because there is still a slow regeneration of 11-*cis*-retinal in the absence of RDH5. Mouse *Rdh5* knockouts do not recapitulate the human retinal phenotype. A cat with spontaneous missense mutation in *RDH5* has been identified and shows key features of the human phenotype with very slow recovery of dark adaptation, evidence of cone dysfunction and degeneration of the macula-equivalent retina region, the area centralis [90]. Figure 3E, F shows fundus images of a human (E) and cat (F) with *RDH5* retinopathy, both species show degeneration of the macula or macula-equivalent area centralis. The missense mutation in the cat was at a well-conserved residue at the site of dimerization, and results in an absence of RDH5 in the affected cat retinal pigment epithelium. Gene augmentation in the cat model shows promise and significantly improves the rate of rod recovery following light-adaptation [91].

## Discussion and future directions

Preclinical therapeutic interventions in animal models are important as part of proof-of-concept and safety studies needed for investigational new drug (IND) approval for human clinical trials. AAV gene therapy approaches for IRDs are the commonest approaches but present certain challenges. One major challenge is that AAVs have a limitation in packing capacity to about ~4.7 kb. This means that some large genes cannot be packaged into a single AAV. Studies to find ways around the AAV packing capacity are well underway using dual or even triple vectors to deliver large therapeutic genes [92]. Another area of active research is to allow for simpler delivery routes than the standard subretinal injection. Intravitreal injection for example can be performed in the office rather than in the operating room required for subretinal injections. However adequate transduction of the RPE and photoreceptors is not achieved by most AAV serotypes when injected intravitreally. Several groups are developing AAV vectors with capsid modifications that aim to give them favourable properties including ability to penetrate to outer retina from intravitreal or suprachoroidal delivery, increased lateral spread of transduction from subretinal injection and to lessen deleterious immune-responses. The cat and dog models offer an opportunity to test these delivery routes and new AAV serotypes in a large eye with the outcome measure being rescue of the disease phenotype.

As a major drawback of retinal gene therapy is the need for mutation specific targeted approaches, canine IRD models can serve for pre-clinical gene therapy trials investigating more generic approaches that if successful would be applicable to many IRDs. Such approaches include optogenetics, whereby a light sensitive protein is expressed on inner retinal cells allowing them to respond to light and trigger messages to the brain [93–95]. Another approach is to introduce neurotrophic proteins or to inhibit expression of proteins involved in cell death [96, 97]. A benefit of gene augmentation therapy in non-dividing retinal cells is its likely permanent presence and effect. However, there may be circumstances where continued expression is not desired. To address this regulatable systems have been investigated, however a favourable outcome of this approach has not yet been achieved in large animal models. Regulatable vectors such as those successfully controlled by doxycycline in mice, have been attempted in dogs, and while they could be regulated, the vectors did not result in vision rescue [98].

While this review focused on the current cat and dog models for which preclinical gene therapy trials have been conducted, there are many additional IRDs that have been documented in these species that could be utilized for future preclinical therapy development. Such examples are the *ABCA4* dog model for Stargardt disease [99] and dogs with *GUCY2D* mutations which mimics LCA1 [100] and cats with a mutation in *CRX* that have a dominant LCA7 like phenotype [101]. Awareness of the availability and potential that these dog and cat models offer is valuable for the greater vision research community offering opportunities to test new promising therapeutic interventions. Furthermore, the dog and cat population provides a potential source of more spontaneous IRD models which may contribute to the development and testing of future treatments for these intractable conditions.
